# Editorial: Exploring lymphocyte signaling: from health to disease

**DOI:** 10.3389/fimmu.2025.1703096

**Published:** 2025-09-24

**Authors:** Rosa Molfetta, Luca Simeoni

**Affiliations:** ^1^ Department of Molecular Medicine, Sapienza University of Rome, Rome, Italy; ^2^ Institute of Molecular and Clinical Immunology, Faculty of Medicine, Otto-Von Guericke University, Magdeburg, Germany

**Keywords:** lymphocytes, signaling, anti-tumor response, immunodefeciency, immunosenescence

Lymphocyte ability to detect and eliminate threats is controlled by an intricate network of signaling factors that are maintained in a delicate equilibrium, ensuring appropriate cellular responses and overall immune homeostasis (1–3). Alterations in these pathways promote tumor escape and immunosenescence (4, 5) and are at the basis of human diseases including immunodeficiency and autoimmunity (6, 7).

The Research Topic *“Exploring Lymphocyte Signaling: From Health to Disease”* aimed to highlight recent developments in the molecular and cellular mechanisms that govern lymphocyte biology. The seven contributions to this Research Topic reflect the wide spectrum of research directions in this field, ranging from receptor cross-talk in natural killer (NK) cells to chromatin remodeling in T cells, immunodeficiency, and strategies to counteract immune aging.

Two contributions emphasize the crucial role of lymphocyte signaling in the battleground in tumor-immune interactions. In their original study, Marangio et al. show that engagement of the NK-cell activating receptor NKG2D not only induces receptor down-modulation but also dampens DNAM-1-mediated signaling, either indirectly through TIGIT upregulation or directly via a mechanism requiring NKG2D endocytosis. These findings show how tumors exploiting NKG2D ligands can broadly impair NK cell cytotoxicity.

In line with this study, a review from Upadhyay et al. highlights the multifaceted role of LFA-1 in anti-tumor immunosurveillance, with a particular focus on NK and T cells. Tumors can subvert this pathway by interfering with adhesion or hijacking downstream signaling, creating an immunosuppressive environment. The article also highlights strategies to restore LFA-1 activity and the importance of LFA-1 in CAR T-cell efficacy, thus underscoring its therapeutic potential.

Beyond signaling mechanisms in lymphocytes in the context of cancer, three contributions address T-cell fate at different regulatory levels involving chromatin remodeling, gene expression, and mRNA splicing. A review by Miao et al. highlights the role of the SWI/SNF chromatin-remodeling complex in lymphocyte differentiation and immune dysfunction. By interacting with transcription factors and shaping chromatin accessibility, SWI/SNF subunits are pivotal for the development of immature lymphocytes and for the function of mature lymphocytes. The review also discusses pathological conditions resulting from subunit loss and the possible use of the SWI/SNF subunits to improve CAR T-cell therapy.

On the other hand, a review by Daniels and Teixeiro highlights the multifaceted role of the NF-κB pathway in T-cell development, differentiation, effector function, and memory, emphasizing how signal integration and signaling dynamics shape these processes. Advances in computational modeling and inducible animal systems may help to resolve how dynamic NF-κB activity influences outcomes *in vivo*.

The third contribution, an original study by White et al., investigates the function of RNA-binding protein hnRNP L in the differentiation of peripheral T cells. Conditional hnRNP L knockout mice displayed normal thymic export but impaired T-cell differentiation into T-follicular helper cells and, accordingly, reduced germinal center responses. It appears that CD4+ T cells lacking hnRNP L failed to differentiate because of a reduced proliferation rate and increased apoptosis, associated with defective upregulation of Bcl-XL. These findings point to a role for hnRNP L in the regulation of alternative splicing of anti-apoptotic factors required to sustain T-cell activation and differentiation.

Collectively, these three articles illustrate that lymphocyte differentiation and function is controlled by intertwined signaling, transcriptional, and epigenetic mechanisms.

Gonzalez-Torbay et al. describe new signaling defects in primary immunodeficiency characterized by a hypomorphic IL2RG variant in a patient with a CVID-like disease. Functional assays revealed reduced STAT3 and STAT5 phosphorylation in CD4_+_ T and B cells, impaired NK-cell degranulation, and defective plasmablast differentiation. This work expands the spectrum of IL2RG-related disease and demonstrates how partial signaling defects underlie heterogeneous clinical manifestations.

Finally, the study of Huang et al. provides a comprehensiveavenues review on T-cell aging and the potential role of telomerase to counteract senescence. Both replicative and premature senescence impair adaptive immunity, thus increasing susceptibility to infection and cancer. By regulating telomere length and other cellular processes, TERT/telomerase may help to restore T-cell functions. The article discusses therapeutic approaches aimed at enhancing telomerase activity as strategies to reinvigorate an aged immune system.

In summary, as shown by the topics of these contributions, regulation of lymphocyte signaling occurs at multiple levels, e.g. receptor activation, transcriptional control, RNA processing, and chromatin remodeling, which reflect the complexity required to maintain an efficient immune function.

This equilibrium can be perturbed by tumors, genetic mutations, or aging, emphasizing the clinical relevance of these regulatory mechanisms. Understanding receptor cross-talk and adhesion pathways is of pivotal importance for improving cancer immunotherapy. Along this line, chromatin remodeling and RNA processing represent novel targets for intervention in T-cell-based therapies. Characterization of hypomorphic variants may help in the diagnosis and management of primary immunodeficiencies. Finally, research on telomerase biology opens a new avenue to counteract immune decline in aging populations.

Future efforts employing integrated approaches and state-of-the-art technologies will strongly deepen our mechanistic understanding and thereby contribute to the development of improved therapeutic strategies.

We thank all authors and reviewers who contributed to this Research Topic. We hope that this Research Topic will serve as a source and inspiration for future studies aimed at understanding lymphocyte signaling in health and disease.
